# “I See a lot of Crazy Things and I Don’t Know What to Believe”: Lessons Learned about Health Literacy and Strategies for Communicating with Vaccine-Hesitant College Students

**DOI:** 10.3390/healthcare11152212

**Published:** 2023-08-06

**Authors:** Corinne N. Berry, Kathleen Walker, Nikole Baker, Claudia Trevor-Wright

**Affiliations:** 1 CommunicateHealth, Rockville, MD 20850, USA; kathleen@communicatehealth.com (K.W.); nikole@communicatehealth.com (N.B.); 2 American College Health Association, Silver Spring, MD 20910, USA; claudiatw@acha.org

**Keywords:** COVID-19, college health, health literacy, health communication, vaccine hesitancy

## Abstract

Throughout the COVID-19 pandemic, the American College Health Association (ACHA) has partnered with CommunicateHealth (CH) to develop COVID-19 mitigation resources for colleges and universities. In 2021, the CH team conducted a series of applied research activities to gain a nuanced understanding of factors that shape perceptions of risk and drive vaccine hesitancy among campus audiences—especially college students who are emerging adults (approximately ages 18 to 22). Based on our findings, CH and ACHA identified key traits of vaccine-hesitant college students and implications for future vaccine communication campaigns. First, vaccine-hesitant students are more likely to ask “why” and “how” questions such as “Why do I need to get vaccinated?” and “How was the vaccine developed and tested?”. Secondly, these students want to have open, authentic dialogue rather than simply accepting health recommendations from a trusted source. Finally, the CH team noted that vaccine-hesitant students were not highly motivated by their own personal risk of getting sick from COVID-19; concern about spreading COVID-19 to others was a much stronger motivating factor. Leveraging these insights, CH and ACHA developed strategies to apply health literacy principles to reach vaccine-hesitant college students with the right information at the right time—and to leverage relevant motivators and overcome barriers to vaccination. By implementing these strategies, CH and ACHA developed clear and empowering educational materials about COVID-19 vaccination tailored to the unique information needs of vaccine-hesitant students.

## 1. Introduction

The COVID-19 pandemic has created unprecedented challenges for public health practitioners and communicators. As the pandemic and simultaneous “infodemic” unfolded in 2020 [1], public institutions—including colleges and universities—made difficult programmatic, policy, and communication decisions in the interest of their communities [2]. To reduce COVID-19 transmission and illness, many campuses across the U.S moved to virtual or hybrid class environments and established social distancing and masking guidelines. Meanwhile, other campuses continued in-person classes and implemented minimal COVID-19 guidelines [3,4].

The United States reached a critical turning point in the pandemic in December 2020, when the Pfizer-BioNTech and Moderna vaccines were granted emergency use authorization by the U.S. Food and Drug Administration (FDA) [5]. A few short months later, in April 2021, the Biden administration announced that all adults were eligible for COVID-19 vaccination [6]. For colleges and universities across the country, the introduction of COVID-19 vaccines brought some hope for a return to “normal” campus life. Yet campus leaders faced another complex, politically fraught decision: should they require COVID-19 vaccination to mitigate transmission? While many campuses required or strongly recommended vaccination, others chose not to implement vaccination policies [7,8]. Campus communicators (staff members whose responsibilities include health education and communication) faced the difficult task of communicating these critical decisions and promoting vaccination to students, faculty, and staff with a wide range of attitudes toward the pandemic and COVID-19 vaccines.

The American College Health Association (ACHA) is charged with advancing the health of college students and campus communities through advocacy, education, and research. Since 2020, ACHA has published COVID-19 guidelines for institutions of higher education (IHE). As public health recommendations have evolved throughout the pandemic, ACHA’s guidelines have provided campus leaders with updates on mitigation strategies like masking, testing, isolation, and vaccination, along with operational and policy considerations for colleges. Early in the pandemic, ACHA also identified a need to provide colleges—particularly those with fewer communication resources—with COVID-19 educational materials and communication strategies. In 2021, ACHA partnered with CommunicateHealth (CH) on the Campus COVID-19 Vaccination and Mitigation Initiative (CoVAC), an initiative to promote COVID-19 vaccines and build vaccine confidence on campuses across the country.

### 1.1. Vaccine Hesitancy and Emerging Adults

Vaccine hesitancy is defined by the World Health Organization as the “reluctance or refusal to vaccinate despite the availability of vaccines” [9].Vaccine hesitancy is a complex phenomenon that has been defined differently over time. Some studies have conceptualized vaccine hesitancy as a continuum of behavior ranging from active demand (low levels of hesitancy) to complete refusal (high levels of hesitancy) [10]. Others have conceptualized and measured vaccine hesitancy as a combination of attitudinal and belief-based constructs that may be held in concert with vaccination behaviors [11].

People may identify different reasons for having concerns about, refusing, or delaying vaccination—and these reasons often differ from vaccine to vaccine [12]. While vaccine hesitancy is not a new concept, it became a more meaningful part of the public consciousness during the 2014 to 2015 outbreak of measles in the U.S., [13] followed by when the World Health Organization declared vaccine hesitancy a “top 10 public health threat”. Much of the empirical research around vaccine-hesitant audiences and communication to address hesitancy has focused on parents [14] because they tend to be primary decision-makers for pediatric immunization.

In a 2020 study on determinants of COVID vaccine hesitancy in the U.S., investigators found that that women, younger adults, individuals who are unemployed, and those with lower socioeconomic status were less likely to get vaccinated for COVID-19 [15]. Additionally, Black Americans reported lower COVID-19 vaccine acceptance than all other racial groups in the study. Results of another national survey [16] found that respondents who self-identified as Republicans were more likely to be less supportive of COVID vaccination, more accepting of misinformation, and less likely to consider COVID a meaningful threat to their family.

Several factors set college students who are emerging adults (approximately ages 18 to 22) apart when it comes to decision-making and information seeking behaviors [17]. During college, many emerging adults learn to make medical decisions on their own for the first time [18]. In addition, these students are more susceptible to peer influence than older adults. As a result, they are more likely to adopt friends’ habits or change their behavior to align with social norms and expectations [19,20]. Finally, emerging adults are more likely to seek out health information on social media platforms like TikTok, Instagram, and Twitter than other age groups [21]. While these platforms can act as valuable tools for self-discovery, expression, and community building, they also enable misinformation and disinformation to spread quickly through global social networks [22,23,24,25].

### 1.2. Study Purpose

This paper outlines a series of applied studies used to inform the development of ACHA’s CoVAC materials: a suite of COVID-19 vaccination resources for colleges and universities. These studies had three specific aims: (1) to identify the priority audience for CoVAC materials, (2) to identify key gaps in existing COVID-19 resources for that audience, and (3) to learn more about the priority audience’s health literacy, knowledge, attitudes, perceptions, behaviors, and communication preferences related to vaccination, so that we could tailor CoVAC materials to meet their needs.

## 2. Methods

### 2.1. Study Design

This study was designed to be exploratory (i.e., not guided by a single theoretical framework) and descriptive to gain a basic understanding of the specific communication context and audience and inform decision-making for the applied communication project. As such, CH conducted three iterative research activities to inform the development of COVID-19 communication materials.

Due to the applied and programmatic nature of this study, the protocols were not submitted to an Institutional Review Board. However, the study team closely followed the ethical principles and guidelines (as outlined in the Belmont Report) [26] including obtaining informed consent from all participants and protecting their privacy by ensuring data were deidentified, password protected, and/or deleted.

Each research activity was intended to build on the previous one and designed with a specific purpose:

- Environmental and literature scan and informal discussions with key informants: Identify existing resources for promoting COVID-19 vaccination and gaps in existing campaigns and materials; ascertain a pulse on national trends in COVID-19 vaccine acceptance and uptake; understand the unmet COVID-19 communication needs of campus audiences.
- Online survey of campus audiences: Understand campus audiences’ COVID-19 information needs and identify the audience segments most likely to express vaccine hesitancy.
- Focus groups: Gain insight into vaccine-hesitant students’ perceptions of COVID-19 vaccination and to test vaccine messages and design concepts.

### 2.2. Environmental and Literature Scan and Key Informant Discussions

The CH team began by conducting an environmental scan of existing COVID-19 resources tailored for college audiences. As part of the environmental scan, we reviewed a variety of resources, including:

- COVID-19 vaccine communication campaign materials;
- COVID-19 educational resources created by CDC, state health departments, and nongovernment public health organizations;
- News articles on campus COVID-19 policies and mitigation strategies from campus newspapers, national college and university organizations, and national news outlets.

We also conducted a literature review of existing vaccine literature and national trend data on risk and vaccine perceptions, especially among various demographics. Findings from this secondary research activity informed the team’s understanding of campus audiences’ attitudes, perceptions, and beliefs about COVID-19 and vaccines and informed development of the survey instrument.

CH also held six informal discussions with campus communicators to supplement findings from the scan. These discussions were 60 min each and were conducted between 18 May through 22, 2021. Key informants were selected based on their role in supporting health promotion and education efforts at their institution. For example, participants included a Director of Nursing and Health Services, student campaign ambassador, vaccine coordinator, and Assistant Director of Wellness Clinic Services.

Findings from the scan and key informant discussions research informed study aims one and two (selection of our priority audience and identifying gaps in the communication landscape). Findings also informed the study design questionnaire development for the subsequent survey and focus groups, which were intended to address study aim three (learning more about the priority audience’s health literacy, knowledge, attitudes, perceptions and behaviors).

### 2.3. Online Survey of Campus Audiences

CH developed and facilitated an online, cross-sectional survey of students, faculty, and staff from colleges and universities across the United States (n = 1583) using Alchemer. From 5 May through 9, 2021, we recruited a non-probability-based sample, aiming for a diverse mix of participants in terms of race and ethnicity, university type and size, and geographic region. The team established quotas to allow for some oversampling of politically conservative respondents, as we had identified conservative political beliefs as a driver of vaccine hesitancy [27].

#### 2.3.1. Questionnaire

The instrument was developed in consideration of key concepts from the Health Belief Model and Theory of Planned Behavior and adapted from Myers and Goodwin, 2011 [28]. Behavioral measures related to vaccination and prevention were adapted from national polls related to COVID-19 [29]. More specifically, the questionnaire included measures of attitude toward vaccination, perceptions of severity and susceptibility, health literacy and knowledge levels, and behaviors related to COVID-19 vaccination and other mitigation strategies (e.g., wearing masks in common spaces on campus).

Vaccine attitude was measured using a series of six semantic differential scales shown to have strong internal consistency (Cronbach alpha 0.97). The item asked whether getting a COVID vaccine would be wise-foolish, worthless-valuable (reverse score), beneficial-harmful, satisfactory-unsatisfactory, bad-good (reverse score). An average of the six items was taken as an overall measure of attitude, with lower scores indicating a more positive attitude.

Perceived susceptibility and severity were measured through three items, each on 7-point Likert scales anchored from “strongly agree” to “strongly disagree”. With an acceptable level of reliability for both measures—0.75 Cronbach alpha for perceived susceptibility and 0.76 Cronbach alpha for perceived severity—an average of the three responses for each item was taken as an overall measure. Lower scores indicated higher levels of susceptibility and severity, respectively.

A single-item health literacy measure that asks respondents “How confident are you at filling out medical forms by yourself?” [30] was used to group respondents into low, medium, or high health literacy categories. The COVID-19 vaccine knowledge item included six statements, five of which were correct and one that was incorrect. The facts were drawn from CDC’s COVID-19 vaccination frequently asked questions page. Respondents were asked to indicate whether each statement was correct or incorrect or select “I don’t know”.

#### 2.3.2. Sampling

Survey respondents were recruited through the Alchemer’s panel to obtain a non-probability-based sample with at least 20% of the sample representing politically conservative audiences. To be eligible to participate, respondents needed to be at least 18 years old and self-identify as a full or part-time student, faculty, or staff member of a U.S. college or university.

The initial sample included 1850 respondents. Before analysis, the CH team cleaned or removed 237 responses based on the amount of time spent on each item (i.e., less than 3 s per item) and any atypical response patterns. The CH team also removed survey responses that were illogical, like nonsensical response options to open-ends (e.g., nonexistent university names) and contradictory responses to multiple items (e.g., younger than 20 with a graduate degree). These revisions to the dataset resulted in a final sample of n = 1583.

#### 2.3.3. Analysis

Using SPSS, we performed descriptive statistical analysis analyzing the frequencies of all variables. As an exploratory study, the intent was to see general characteristics of the audience surveyed, breadth of responses among certain variables, obtain feedback on message concepts for the campaign, and identify any areas for further exploration in qualitative activities. Using SPSS 27 Custom Tables, we created cross-tabulations for our exploratory inferential analysis of key variables in the survey. We applied the Bonferroni correction to adjust all pairwise comparisons and then identified significant differences between proportions based on 2-sided tests at the 0.05 significance level.

### 2.4. Focus Groups with Vaccine-Hesitant Students

From 8 June through 9 July 2021, CH conducted focus groups with vaccine-hesitant students. We identified vaccine-hesitant students based on their response to the screening question “When a COVID-19 vaccine is available to you, what will you do?” We categorized students who selected the following response options as vaccine-hesitant:

- Wait until it has been available for a while to see how it is working for other people.
- Only get the vaccine if required for work, school, or other activities.
- I have not decided.

#### 2.4.1. Recruitment

Based on findings from national poll data [31], we focused recruiting efforts on individuals who identified as politically conservative, Hispanic or Latinx, and Black or African American—three groups that were more likely to express vaccine hesitancy at the time of this study. We conducted nine focus groups (n = 47)—three for each audience segment—to explore students’ perceptions of the COVID-19 vaccines and test the effectiveness of draft messages promoting COVID-19 vaccination.

CH enlisted a professional recruitment firm to recruit and screen individuals to participate in the focus groups. All focus groups were scheduled with one over-recruit to ensure at least five participants per group. The participant screener and recruitment process focused on obtaining a diverse mix of vaccine-hesitant individuals, including gender, geographic region, and age, and minimizing participation of vaccine-resistant individuals.

#### 2.4.2. Discussion Guide

The discussion guide was written to align with the theoretical concepts explored in the survey but in greater depth and nuance. The questions and prompts were open-ended and used as a framework to guide each group’s conversation. The discussion began with questions about their understanding and perceptions of risk of COVID. This was followed by questions about their current knowledge, attitudes, and information sources related to the vaccine. The final segment provided opportunity for students to respond to a variety of messages about the COVID-19 vaccine.

#### 2.4.3. Analysis

At the conclusion of the focus groups, all transcripts were reviewed and initially coded by the lead researcher. A secondary researcher reviewed the field notes and captured key takeaways. The research team discussed preliminary codes and aligned on coding schema. Additional reviews of transcripts were conducted to identify conceptual connections and/or emerging themes.

### 2.5. The COVID-19 Landscape during This Research

Because COVID-19 vaccines had only been widely available for a short time when we conducted the survey, uncertainty, apprehension, and speculation about the vaccines were at an all-time high. During the same week, the Centers for Disease Control and Prevention (CDC) also lifted indoor masking recommendations for fully vaccinated individuals [32]. For many Americans, CDC’s announcement signaled a new stage of the COVID-19 pandemic, a significant departure from pandemic restrictions that marked 2020 and continued into 2021 in some areas of the country. In addition to these recent shifts, emerging public awareness of COVID-19 variants may have shaped participants’ views on the pandemic. When we fielded focus groups in summer 2021, the Delta variant accounted for most COVID-19 cases among unvaccinated Americans [33]. Rapid shifts in masking guidance also contributed to public confusion about COVID-19 mitigation strategies and their effectiveness. Just two months after lifting indoor masking recommendations (and a few weeks after our focus groups concluded), in July 2021, the CDC again recommended that vaccinated individuals wear masks indoors [34]. These shifts contributed to sentiments that federal agencies were mismanaging the pandemic, withholding key information, or even actively manipulating the American public [35]. In short, a variety of factors, including the novelty of vaccines, the emergence of variants, and shifting recommendations on masking may have informed participants’ perceptions of COVID-19, vaccination, and individual and collective responsibility to mitigate transmission of the virus.

## 3. Results

### 3.1. College Audience Segments and Vaccine Readiness

The CH team’s survey results suggested that respondents could be categorized into three groups related to their attitudes and perceptions of COVID-19 vaccines: vaccine-ready, vaccine-hesitant, and vaccine-resistant. The vaccine-ready group included people who had already received at least one dose of a COVID-19 vaccine, as well as people who said they planned to get vaccinated “as soon as possible”. At the other end of the spectrum, the vaccine-resistant group said they would “definitely not” get vaccinated.

The vaccine-hesitant group fell between those two extremes, encompassing a range of responses. Some people in this group said they would only get a COVID-19 vaccine if their campuses required vaccination. Others said they would “wait and see”. As one associate dean of student engagement explained, “People…are kind of on the fence and are waiting to see what will happen with the vaccine. They want more info about data and how people are going to react—6 months, a year, maybe a little longer—to the vaccine”. Other respondents in the vaccine-hesitant group said they did not know if they would get a COVID-19 vaccine or declined to answer the question.

The survey data revealed the following about each segment:

- Individuals in the vaccine-ready group stated that they had already received a COVID-19 vaccine or planned to do so as soon as possible. Therefore, we determined that this group was most likely to complete the desired action step (getting a COVID-19 vaccine) without meaningful intervention or encouragement.
- The vaccine-resistant group showed a high level of resistance to COVID-19 vaccination. In comparing these data with national trends, we determined that these individuals were likely more fixed in their attitudes and perceptions of COVID-19 vaccination and would be less open to influence from a health communication campaign [36].
- Findings related to the vaccine-hesitant group suggested that these individuals might be open to the possibility of getting vaccinated but wanted more information or credible reassurance about the safety and efficacy of the vaccines. It is noteworthy that vaccine-hesitant respondents in the survey were significantly less likely to report high health literacy levels (66%) compared to vaccine-ready respondents (76%). Based on these insights, ACHA and CH decided to focus the development of communication resources to support decision-making among the vaccine-hesitant audience.

### 3.2. The “Vaccine-Hesitant” Priority Audience

A total of 1583 students, faculty, and staff completed the survey, and we identified 338 individuals as vaccine-hesitant based on their responses. Of the students, faculty, and staff in the vaccine-hesitant group,

- 50% were 18 to 24 years old;
- 80% were students;
- 68% indicated they had never had COVID-19;
- 72% indicated their family and friends were already vaccinated;
- 41% were from the Southern or Southwestern regions of the US (by comparison, 24% of vaccine-ready individuals were from the South or Southwest).

In all, 40% to 54% of the vaccine-hesitant respondents indicated that they “always” engage in COVID-19 mitigation behaviors like wearing a face mask and avoiding non-essential travel. By comparison, only 23% to 26% of vaccine-resistant respondents reported engaging in these protective behaviors.

In all, 17% of vaccine-hesitant respondents indicated that they believe they had contracted COVID-19 at some point during the pandemic, but they were never officially diagnosed by a health care professional. By comparison, 12% of vaccine-ready respondents reported the same.

In all, 44% of vaccine-hesitant respondents expressed that at least one acquaintance had contracted COVID-19, but the acquaintance did not experience severe symptoms. By comparison, 32% of vaccine-resistant respondents reported the same.

### 3.3. Vaccine Hesitant Audience: Perceptions of Risk

Vaccine-hesitant respondents tended to acknowledge the severity of COVID-19 but reported mixed perceptions of their susceptibility to COVID-19. A total of 79% of vaccine-hesitant respondents agreed with the statement “Complications from COVID-19 are serious”. By contrast, only 58% agreed with the statement “Getting COVID-19 is currently a possibility for me”, and 45% agreed with the statement “I will be very sick if I get COVID-19”. Only 34% agreed with the statement “My chances of getting COVID-19 in the next few months are high”.

Despite these lower perceptions of their own COVID-19 risk, the majority of vaccine-hesitant respondents (75%) indicated they were worried about transmitting COVID-19 to someone else without realizing it—significantly more than vaccine-resistant respondents (54%), but significantly less than vaccine-ready respondents (83%).

### 3.4. Vaccine Hesitant Audience: Knowedge, Health Literacy, and Information Needs

More than two thirds of vaccine-hesitant survey respondents (71%) answered half or fewer of the survey’s COVID-19 knowledge items correctly. On some key questions related to common vaccines myths and misconceptions, a significant number of respondents selected “I don’t know”. For example, in response to the statement “COVID-19 vaccines cannot make you sick with COVID-19”, 35% responded “I don’t know”. To the statement “COVID-19 vaccines do not change or interact with your DNA”, 42% responded “I don’t know”. These responses indicated that hesitant audiences were lacking an understanding of key facts about COVID-19 and the vaccine.

When asked what they wanted to know about COVID-19 vaccination, the focus group participants focused on side effects of COVID-19 vaccination—including long-term health problems and short-term issues like allergic reactions. One participant in a Black/African American focus group explained, “I would like to know the long-term side effects, but that’s not an answer I can get in the foreseeable future. I think that’s what makes it really hard”. A few participants asked about the ingredients in the COVID-19 vaccines.

The survey and focus groups also offered an opportunity for CH and ACHA to learn more about COVID-19 vaccine myths, misconceptions, and mis- and disinformation that shaped campus audiences’ perception of the vaccines. When asked “What, if anything, have you seen or heard about the COVID-19 vaccine that you know to be false or untrue?” participants shared statements that ranged from common misconceptions to conspiracy theories, including the following examples:

- The COVID-19 vaccine causes COVID-19;
- The COVID-19 vaccine causes severe side effects—like infertility or reproductive problems in women;
- The COVID-19 vaccine changes your DNA;
- The COVID-19 vaccine uses aborted fetuses in manufacturing.

In focus groups, participants shared examples of misinformation circulating in their social networks. Reproductive health consistently emerged as a concern for vaccine-hesitant individuals, especially cisgender female students. One student in a politically conservative focus group reported, “I’ve heard from multiple people that I know who have gotten the vaccine, that were pregnant at the time and miscarried”. When asked what vaccine-related topics they would like to learn more about, several respondents said they wanted more information about fertility or women’s reproductive health.

One focus group participant shared, “Recently there’s this video that has gone viral. You see people placing [magnets] on their shoulders on the place where they got the shot. They place a lightbulb where they got the vaccine, and you see the lightbulb just ignites. I see a lot of crazy things and don’t know what to believe”. Another participant stated, “On Facebook, people always be like ‘Don’t get the shot because it made my eyes swell up’. …I don’t want my eye to be swollen, so that gives me some apprehension about getting the shot”. Though students characterized these social media posts as misinformation, by concluding their accounts of such posts with statements like “I don’t know what to believe” and “that gives me some apprehension”, they conveyed a sense of uncertainty about whether the claims could possibly be true.

### 3.5. Vaccine Hesitant Audience: Attitudes and Perceived Norms

In the survey, respondents were asked to rank their attitude about COVID-19 vaccines using a 7-point semantic differential scale. The question was phrased as “I think that getting a COVID-19 vaccine is…” with response options ranging from wise to foolish, valuable to worthless, beneficial to harmful, satisfactory to unsatisfactory, good to bad, and positive to negative. Lower scores indicated a positive attitude toward COVID-19 vaccines. Overall, the scores confirmed our expectations about vaccine readiness and attitudes toward COVID-19 vaccines. On average, vaccine-hesitant respondents scored 3.55, indicating they were more neutral or undecided in their attitudes about COVID-19 vaccines. By contrast, the vaccine-ready segment averaged 1.83, indicating more positive attitudes, and the vaccine-resistant segment averaged 5.61, reflecting more negative attitudes toward vaccination.

In the focus groups, participants expressed concerns about how quickly the vaccines were developed, making connections between the relatively rapid vaccine development process and the possibility of side effects or long-term health concerns. Participants’ word choices also highlighted uncertainty as a primary driver of hesitancy. The most common words participants used when sharing their thoughts on the COVID-19 vaccines were related to mistrust and risk (e.g., “unpredictable”, “risky”, “untrustworthy”, “skeptical”).

When survey respondents were asked to reflect on what their close circle of family or friends and family,

- Only 5% of vaccine-hesitant respondents said they thought “everyone” in their close circle would get a COVID-19 vaccine, while 38% said they thought “most” people would get vaccinated;
- 31% said “about half” of their circle would get vaccinated;
- 17% said “very few” people would get vaccinated, and 4% said “no one” would get a COVID-19 vaccine.

The focus group participants expressed concerns that the COVID-19 vaccine could potentially cause health problems in the future, and uncertainty surrounding the new vaccines magnified those concerns. For example, one participant stated, “I’m 19 years old, I’m healthy to my knowledge, and I’ve seen friends get it [COVID-19], and they didn’t even know they had it. So, I’d almost just rather get it [COVID-19] than have something foreign put in my body that quite frankly doesn’t need to be there”.

### 3.6. Vaccine Hesitant Audience: Information Sources

When asked whom they had talked to about getting vaccinated, most focus group participants mentioned having conversations about the COVID-19 vaccines with family members. Some participants stated that their parents were against the vaccines and were pressuring them not to get vaccinated. By contrast, some participants in the Black/African American and Hispanic/Latinx focus groups mentioned that extended family members were pushing them to get vaccinated, with a few mentioning pressure from the elders in their family.

In in-depth interviews, campus communicators echoed students’ sentiments about parental influence. One staff member in a university health services office stated, “We had a student who got vaccinated.… We received a letter from an irate parent who couldn’t believe the university let her daughter get vaccinated and she was going to hold them accountable if anything happened to her daughter [because of] the vaccination. She was adamant about holding the university responsible because [she said] the vaccine isn’t safe”. A COVID-19 project manager at another university explained, “It is a huge thing for students that their parent is like ‘I’m not so sure,’ and so then they don’t want to go against their father and get the vaccine”.

Many students in the focus groups mentioned having conversations about the COVID-19 vaccines with friends, significant others, and coworkers. Some explained that conversations about the COVID-19 vaccines had become divisive, as their conversation partners expressed strong opinions for and against the vaccines. In addition to the influence of their social circles, focus group participants shared that they got information about COVID-19 vaccines from social media, naming channels like TikTok, Instagram, Facebook, Snapchat, and YouTube. Social media algorithms can create echo chambers that reinforce students’ beliefs and perceptions—potentially amplifying the impact of misinformation and disinformation.

When asked how they determined if an online source was credible or trustworthy, some said they had trouble deciding what information or sources to believe. For example, one participant stated, “You can’t tell what’s real or not”. Some mentioned taking steps to make sure information was credible or trustworthy, like searching on Google, looking at other data or research, and looking at verified posts on social media. Only one student mentioned using the CDC website (cdc.gov) to confirm COVID-19 information.

### 3.7. Vaccine Hesitant Audience: Barriers and Motivators

Survey results pointed to some specific beliefs and perceptions that served as barriers to vaccination among the vaccine-hesitant group. When asked, “Which of the following statements about the COVID-19 vaccine are true for you?”, 72% of vaccine-hesitant respondents agreed with the statement “I am concerned about possible side effects”. Nearly half of vaccine-hesitant respondents also agreed with the following statements:

- I do not know if the COVID-19 vaccine works (45%);
- I need more information first (43%);
- I am concerned about having an allergic reaction (42%).

In addition, 28% of vaccine-hesitant respondents agreed with the statements “I don’t trust COVID-19 vaccines” and “I think other people need it more than I do right now”.

Many focus group participants expressed concerns about how quickly the vaccines were developed, making connections between the relatively rapid development process and the possibility of unknown, long-term health concerns. One participant captured this popular sentiment and expressed skepticism about media promotion of the vaccine, stating, “It’s new and uncertain—we don’t know what will happen in the long run.... I feel like it was pushed out very fast, and I’ve never seen a vaccine get as much screen time and advertisement, especially with it being so new and not seeing the long-term effects”. One focus group participant echoed the concerns about future health effects, sharing, “We’re not going to know the 30-year side effects until 30 years from now, but does it matter if COVID gets you first? And so, it’s like either you subject yourself to the powers that be as far as COVID, or you take chance on the vaccine, which could be harmful or helpful”.

When asked, “Which of the following reasons to get a COVID-19 vaccine are true for you?”, vaccine-hesitant respondents were most likely to agree with the statement “To protect others from getting sick”, with 56% of respondents selecting this statement. Nearly half of respondents also agreed with following statements:

- To protect myself from getting sick (48%);
- To help stop the virus/pandemic (48%);
- To get life back to normal (42%).

Comparatively fewer respondents agreed with the statements “To be able to travel” (29%) and “Because I believe in vaccines and science” (24%).

### 3.8. Vaccine Hesitant Audience: Responses to Messages

As part of the survey, the CH team gathered respondents’ feedback on a number of sample messages related to COVID-19 vaccination. CH developed these sample messages based on barriers to vaccination identified during our literature review, leveraging plain language and health literacy best practices. We utilized messages from a March 2021 Kaiser Family Foundation survey and vaccine confidence messaging recommendations from the Ad Council’s COVID Collaborative COVID-19 Vaccine Education Initiative as a starting point in the message development process.

Vaccine-hesitant respondents indicated that they felt “confident or extremely confident” about getting vaccinated after reading the following messages:

- The vast majority of U.S. doctors who are eligible to get a COVID-19 vaccine have taken the vaccine (29% of respondents).
- It is true that most people who die from COVID-19 are older or have other health problems. But many young and healthy people have also died or developed serious health problems because of COVID-19 (25%).
- It is true that the COVID-19 vaccines are new. But scientists have been working on the technology used in the vaccines for 20 years (24%).
- It is very common to feel tired and achy or run a low fever for a few days after getting a COVID-19 vaccine. But these side effects only last for a short time. They are signs that your body is building up protection—and that means the vaccine is working (23%).
- Even if you have already had COVID-19, you can still get it again. That is why it is important for everyone to get a COVID-19 vaccine (23%).

Based on survey results, the CH team eliminated, expanded, or further tailored messages for testing with vaccine-hesitant students in focus groups.

Table 1 below provides findings from each of the messages tested in focus groups.

## 4. Discussion

### 4.1. Implications for Health Literacy

According to Healthy People 2030 [37], personal health literacy is defined as “the degree to which individuals have the ability to find, understand, and use information and services to inform health-related decisions and actions for themselves and others”. The information landscape has undergone tremendous change throughout the pandemic, posing new challenges for consumers to find, understand, and use meaningful (and factual) health information. Health literacy is often conceptualized as a product of one’s situational state rather than fixed a trait [38]. Thus, anyone can experience limited health literacy at some point in their life—especially when making decisions in a novel situation based on complex health and risk information. College students and campus audiences are no exception to this rule.

The results of this study shed some light on a potential relationship between health literacy and vaccine hesitancy—an area that is ripe for further exploration. These results support the idea that people at all levels of health literacy and vaccine hesitancy can and often will make the choice to be vaccinated. However, consistent with previous research [39,40], this decision is not always made with confidence or positive emotions. Additionally, the decision to get vaccinated is often driven by a variety of internal and external factors. This research suggests that people with higher health literacy may have an easier time identifying vaccine misinformation and are attitudinally positioned to accept, and feel confident about, simple factual messages about COVID-19 vaccination. Those with moderate and lower levels of health literacy experience greater difficulty processing vaccine information, feel social pressure to be vaccinated, and may find basic vaccine messages insufficient to meet their information needs and build confidence. While it is noteworthy that participants with low to moderate health literacy held significantly more negative attitudes toward COVID-19 vaccination than those with high health literacy, there is much to be uncovered regarding when and how this attitude formation occurs.

### 4.2. Finding Health Information

Consistent with recent findings [41], the CH team’s research revealed that vaccine-hesitant campus audiences (primarily students) were exposed to a great deal of COVID-19 and vaccine mis- and disinformation—particularly through social media channels. To further complicate matters, this exposure typically occurred as a result of incidental scrolling through social feeds as opposed to intentional information search. Little is known about how people “attend” to misinformation when exposed incidentally versus if they come across it when conducting an active search. However, marketing and branding research suggests that incidental social media exposure can unknowingly influence consumers’ behavior [42]. It is plausible that as consumers, we may be more likely to question the credibility of information when we purposefully seek out that information. In fact, research has demonstrated that pre-bunking, or warning people that misinformation may be ahead, is a useful strategy in immunizing audiences against the impact of false information [43].

The increasingly complex and politically polarized information environment at the time of our research may have influenced students’ levels of motivation to seek out accurate information about COVID-19 vaccines. Our research revealed that students relied heavily on their parents and family members in assessing the veracity of COVID-19 vaccine information and ultimately making the decision to get vaccinated (or to avoid COVID-19 vaccination). As many emerging adults are navigating health decisions independently for the first time, these students turn to trusted elders to verify health information and get guidance to inform their decisions.

### 4.3. Understanding Health Information

Vaccine-hesitant focus group participants acknowledged having information gaps related to the COVID-19 vaccines. In conveying their questions and concerns about the vaccines, participants highlighted how a lack of scientific certainty presents challenges in assessing personal and community risk, saying the that vaccines were new, not fully proven by research, and came out too quickly. Seeking out and understanding credible scientific information about COVID-19 vaccination proved a complex task. By contrast, our focus group participants were inundated with relatable, compelling, and easy-to-understand anecdotes about COVID-19 vaccination through their social networks. Students may have questioned the veracity of these claims—like a friend of a friend’s social media post about menstrual changes or a parent’s account of missing a full week of work after getting vaccinated—but the accounts still contributed to their vaccine hesitancy. Ultimately, because these anecdotes were easy to understand, they were also easy to believe, regardless of their accuracy.

### 4.4. Using Health Information

Vaccine-hesitant focus group participants used information in a variety of ways when making a decision about getting vaccinated. While participants generally expressed a dislike for COVID-19 vaccine requirements at their school, some indicated they would get vaccinated if it was required by their school or job.

Other focus group participants mentioned discussing the pros and cons of getting vaccinated with people they trusted, like friends and family. These focus group participants acknowledged that their family members’ experiences and beliefs factored into their decision-making because they felt pressured one way or another. This is consistent with the findings from our survey and interviews with key informants. Vaccine-hesitant respondents in our survey were significantly more likely to feel social pressure to get the COVID-19 vaccine, compared to those who were vaccine-resistant. Anecdotally, key informant interview participants reflected on how getting a COVID-19 vaccine was the first big medical decision students had to make—and when parents advised students not to get vaccinated, students tended to take their advice.

### 4.5. Implications for Health Communication

As the COVID-19 pandemic continues to evolve, many public health professionals have expanded their focus from the acute need to promote COVID-19 vaccination to the larger task of building vaccine confidence. This work is critical to building a foundation for successful emergency response. Based on our findings, the CH team identified key traits of vaccine-hesitant audiences and implications for future vaccine communication campaigns.

First, vaccine-hesitant audiences are more likely to ask “why” and “how” questions such as:

Why do I need to get vaccinated?

How was the vaccine developed and tested?

How do we know that the vaccine works?

How do we know if the vaccine could cause side effects or long-term health problems?

By addressing these questions explicitly, health communicators can build trust, signal transparency, and meet vaccine-hesitant audiences where they are.

The CH team learned that vaccine-hesitant readers want communication materials that feel open, honest, and authentic. They want to have a conversation or dialogue rather than simply accepting recommendations from a trusted source. Health communicators can create space for this dialogue by acknowledging scientific uncertainty, shifts in public health guidance, or competing viewpoints on an issue. Incorporating personal story content, such as quotes from individuals explaining why they made the decision to get vaccinated, can also help to create a sense of authentic conversation in vaccine communication materials.

Finally, the CH team noted that vaccine-hesitant audiences were not highly motivated by their own personal risk of getting sick from COVID-19. Concern about spreading COVID-19 to others was a much stronger motivating factor. Based on this finding, we recommend that vaccine communication materials emphasize community belonging and incorporate messages about protecting others. To enhance emotional appeal, these messages can focus on specific people or groups of concern to the priority audience. For example, in the context of the COVID-19 vaccination materials for college students, the CH team incorporated messages about getting vaccinated for a grandparent or a favorite professor.

The CH team leveraged these insights to develop a suite of communication materials for vaccine-hesitant students, including social media graphics, posters, digital signs, and other materials for colleges and universities to disseminate on campus. We crafted messaging to align with students’ drive to express personal autonomy in health decisions and motivation to protect others from getting sick. The resulting messages frame vaccination as a personal choice and emphasized reasons why students might choose to get vaccinated. For example, social media graphics featured testimonial-style messages like “I got vaccinated for my favorite professor” and “I got boosted to protect the people who’ve always been here for me”, paired with relatable stock photos of young adults and a clear call to action to stay up to date on COVID-19 vaccines. Health communication materials can use this approach to promote other vaccinations and mitigation behaviors while honoring audiences’ capacity for personal decision-making.

### 4.6. Limitations and Future Research Directions

The non-probability survey may not be entirely representative of the larger U.S. college and university population. The CH team initially oversampled demographic groups that were known to express higher levels of vaccine hesitancy at that time. Future survey research could provide additional opportunities to explore drivers of vaccine hesitancy among emerging adults and greater insight into relationships that may exist between health literacy and hesitant attitudes or beliefs.

Due to timing constraints, the research activities described above coincided with the resource development phase of our partnership with ACHA. This limited the number of messages we could test and refine based on participants’ feedback. As noted in Section 2.5, the rapid pace at which scientific knowledge, public health guidance, and media messaging about COVID-19 evolved at the time of our research likely impacted participants’ perceptions and attitudes toward COVID-19 vaccination. During periods of high uncertainty, health communicators might consider options for conducting rapid, iterative message testing to ensure sufficient tailoring for each relevant audience.

The scope of this applied project only allowed for basic quantitative analyses. Future studies should be designed for more robust inferential analyses and consider the usage of cluster analysis for the purpose of audience segmentation.

Beyond the limitations of this study, future research related to vaccine confidence on campus could explore the following topics:

- How students make decisions about vaccines, since deciding whether to get vaccinated may be the first big health decision that emerging adults make independently;
- How campus COVID-19 vaccine policies or requirements impacted vaccine confidence among students;
- How factors specific to each campus’s culture and community affected COVID-19 vaccine uptake. These factors might include a robust presence of health services on campus, high levels of trust in campus administration, affiliation with a medical center, and an existing ethos of community responsibility on campus.

## 5. Conclusions

From our research with campus audiences, the CH team identified key insights related to health literacy and applied health communication. Campus audiences faced an increasingly complex and polarized information landscape as they navigated novel health decisions during a global public health emergency. Adding to the complexity, many students are at a developmental stage where they are learning to make health decisions independently for the first time. This research highlighted some important communication considerations for promoting vaccine confidence among college students, including social influence from peers and family members, social media usage (specifically the exposure to misinformation), and fluctuating perceptions of COVID-19 risk. These findings also underscored the importance of using a tailored approach to communicating with vaccine-hesitant students—where information gaps are filled, there is sufficient level of explication, and where messages convey empathy and use salient motivators. CH and ACHA leveraged these insights to create effective and engaging vaccine communication materials for colleges and universities across the country, with a focus on vaccine-hesitant student populations.

## Figures and Tables

**Table 1:** Student feedback on COVID-19 vaccine messages.

Message	Student Feedback
“Do you have questions about COVID-19 vaccines? Don’t be afraid to ask! The campus health center can help you make an informed decision”.	About half of the focus group participants said they liked this message because it encouraged them to make an informed decision. Participants pointed out that the message framed COVID-19 vaccination as a conversation and a choice, noting that they want to learn about the COVID-19 vaccines and see medical professionals as a trusted source of information.
“You can’t get COVID-19 from a vaccine. You might feel tired and achy after getting vaccinated, but these side effects are a sign that the vaccine is working—not symptoms of COVID-19”.	Some participants said this message was most believable because they had experienced side effects after other vaccines. Some mentioned that they knew people who had side effects after getting a COVID-19 vaccine. A couple of participants described this message as “more honest” than the others because it acknowledged a downside of getting vaccinated.
“Researchers haven’t found any long-term side effects from COVID-19 vaccines. But they have found long-term health problems from getting COVID-19—even in people who didn’t have any symptoms when they were first infected with COVID-19. Experts agree that it’s much safer to get vaccinated than to risk getting sick with COVID-19”.	Some students said this message was “motivating” because it was honest about the possibility of side effects, and one participant described the message as “caring”. A few participants stated that the message made them feel safer and more prepared for the vaccine’s side effects. Others said they disliked the message because we can’t know if the COVID-19 vaccines will have long-term side effects.
“The COVID-19 vaccines were developed more quickly than previous vaccines. The process was fast because international researchers, scientists, and government agencies worked together to put an end to the pandemic. The paperwork was fast-tracked—but the clinical trials were not”.	Many students in the Hispanic/Latinx focus groups said it was “motivating” to think about so many people and agencies working together worldwide. A few participants in the Black/African American focus groups said they liked the message about fast-tracking the vaccine, but a couple said they did not find the message motivating because it stated that other trials had been going on for 20 years.
“There’s no evidence that getting a COVID-19 vaccine can cause fertility problems. There’s been confusion about this because of a false report that spread on social media. The report said that getting a COVID-19 vaccine could cause a woman’s body to attack the wrong spike protein—one that’s related to pregnancy. But the spike protein on the coronavirus and the one related to pregnancy are completely different, and your body knows that”.	At least one participant said the information in this message put them at ease. However, others had a strong negative reaction, stating that there is no proof that the vaccine does not cause miscarriages. Some reported that they had heard of studies and personal stories about miscarriages or fertility problems after getting a COVID-19 vaccine.

## Data Availability

The data presented in this study are available on request from the corresponding author. The data are not publicly available due to privacy and ethical restrictions.
